# Pseudorabies virus hijacks the Rab6 protein to promote viral assembly and egress

**DOI:** 10.1186/s13567-024-01328-4

**Published:** 2024-05-28

**Authors:** Dong-Ge Liang, Yu-Kun Guo, Shi-Bo Zhao, Guo-Yu Yang, Ying-Qian Han, Bei-Bei Chu, Sheng-Li Ming

**Affiliations:** 1 College of Veterinary Medicine, Henan Agricultural University, Zhengzhou, 450046 Henan China; 2 Animal Diseases and Public Health Engineering Research Center of Henan Province, College of Food and Drugs, Luoyang Polytechnic, Luoyang, 471023 Henan China; 3 Key Laboratory of Animal Biochemistry and Nutrition, Ministry of Agriculture and Rural Affairs, Zhengzhou, 450046 Henan China; 4 Key Laboratory of Animal Growth and Development of Henan Province, Henan Agricultural University, Zhengzhou, 450046 Henan China; 5 Longhu Advanced Immunization Laboratory, Zhengzhou, 450046 Henan China; 6 International Joint Research Center of National Animal Immunology, Henan Agricultural University, Zhengzhou, 450046 Henan China; 7 Ministry of Education Key Laboratory for Animal Pathogens and Biosafety, Zhengzhou, 450046 Henan China

**Keywords:** PRV, small GTPase, Rab6, gB, gE, viral assembly and egress

## Abstract

Pseudorabies virus (PRV) is recognized as the aetiological agent responsible for Aujeszky’s disease, or pseudorabies, in swine populations. Rab6, a member of the small GTPase family, is implicated in various membrane trafficking processes, particularly exocytosis regulation. Its involvement in PRV infection, however, has not been documented previously. In our study, we observed a significant increase in the Rab6 mRNA and protein levels in both PK-15 porcine kidney epithelial cells and porcine alveolar macrophages, as well as in the lungs and spleens of mice infected with PRV. The overexpression of wild-type Rab6 and its GTP-bound mutant facilitated PRV proliferation, whereas the GDP-bound mutant form of Rab6 had no effect on viral propagation. These findings indicated that the GTPase activity of Rab6 was crucial for the successful spread of PRV. Further investigations revealed that the reduction in Rab6 levels through knockdown significantly hampered PRV proliferation and disrupted virus assembly and egress. At the molecular level, Rab6 was found to interact with the PRV glycoproteins gB and gE, both of which are essential for viral assembly and egress. Our results collectively suggest that PRV exploits Rab6 to expedite its assembly and egress and identify Rab6 as a promising novel target for therapeutic treatment for PRV infection.

## Introduction

Pseudorabies virus (PRV), a member of the alphaherpesvirus family, possesses a genome approximately 143 kb in size and contains at least 70 open reading frames [1]. PRV, also known as porcine pseudorabies virus or porcine herpes virus, is a significant infectious agent affecting pigs and several other mammalian species [2]. Since 2011, there has been a notable increase in the prevalence of PRV among the pig population in China. This increase is attributed to the emergence of PRV variants that exhibit an enhanced ability to evade the immune protection conferred by existing commercial vaccines [3, 4]. PRV is a herpesvirus, and it has caused considerable economic losses in the livestock and animal husbandry sectors. Furthermore, it represents a potential risk to human health [5–7]. Despite the availability of some vaccines and control strategies, the prevention and management of PRV infection still present numerous challenges. There is a pressing need for continued research into the pathogenic mechanisms of PRV. Such endeavors are expected to provide new theoretical groundwork for developing more effective strategies to combat PRV infection.

Rab proteins are a large family of small GTPases that belong to the Ras superfamily [8]. Their pivotal functions are integral to the control of vesicle trafficking, involving vesicle formation, movement, and fusion [9]. With over 60 known members in humans alone, the RAB family displays a diverse range of localizations and functions specific to each member [10]. A multitude of viruses have evolved mechanisms to hijack RAB proteins, manipulating the host’s intracellular transport systems to bolster their own replication and dissemination [11]. For example, rabies virus leverages interactions with RAB5 to hijack the host cell’s endocytosis pathway, facilitating cell entry and further infection [12]. Furthermore, hepatitis C virus is known to utilize several RAB proteins, such as RAB5, RAB7, and RAB11, to aid in its replication and assembly processes [13]. In cells infected with herpes simplex virus type 1 (HSV-1), RAB27a-positive vesicles facilitate the transport of virus particles to the cell periphery, thus enhancing virus transmission [14]. Additionally, Rab18 has been identified as a promoter of dengue virus infection by directing fatty acid synthase to sites of viral replication [15]. Interestingly, Rab6 has been implicated in the transport of viral glycoproteins to facilitate envelope assembly during HSV-1 infection [16]. However, the specific role of Rab6 in the life cycle of PRV has not been fully determined.

In our research, we focused on revealing the influence of Rab6 on PRV replication. Our findings revealed that PRV exploits Rab6 to facilitate viral assembly and egress, highlighting the pivotal role of Rab6 in the propagation of PRV infection. This insight not only advances our understanding of PRV pathogenesis but also highlights new avenues for developing targeted antiviral therapies to disrupt the interaction between PRV and Rab6.

## Materials and methods

### Reagents

TRIzol Reagent (D9108B) and SYBR Premix *Ex Taq* (RR420A) were obtained from TaKaRa; anti-Rab6 (10187-2-AP), anti-HA (51064-2-AP), and anti-FLAG (66008-4-Ig) antibodies were purchased from Proteintech. Antiserum against the PRV glycoproteins gB and gE were generated by immunizing mice with purified recombinant gB and gE.

### Cells, viruses and plasmids

Porcine kidney epithelial PK-15 (CCL-33, ATCC), Vero (CRL-1586, ATCC), porcine alveolar macrophages (PAM [17]), and HEK293T (CRL-11268, ATCC) cells were grown at 37 ℃ with 5% CO_2_ in DMEM (12110, Solarbio) supplemented with 10% foetal bovine serum (FBS, A5669701, Gibco), 100 U/mL penicillin and 100 μg/mL streptomycin sulfate (B540732, Sangon).

The virulent PRV isolate QXX (PRV-QXX) was kindly donated by Yong-Tao Li from the College of Veterinary Medicine, Henan Agricultural University [18]. The recombinant PRV strain PRV-GFP was derived from the PRV Hubei strain, with the TK gene replaced by a GFP expression cassette from the pEGFP-N1 plasmid [19].

Full-length porcine Rab6 cDNA was amplified by polymerase chain reaction (PCR). Rab6 cDNA was cloned and inserted into p3 × FLAG-CMV-10 expression plasmids to generate FLAG-Rab6, FLAG-Rab6 Q72L and FLAG-Rab6 T27N. All plasmids were transfected with Lipofectamine 3000 (L3000015, Invitrogen) according to the manufacturer’s instructions. The primer sequences used for gene amplification were as follows: Rab6-Fw: 5ʹ-ATGTCCACGGGCGGAGAC-3ʹ; Rab6-Rv: 5ʹ-TTAGCAGGAACAGCCTCCTTCA-3ʹ; Rab6 Q72L-Fw: 5ʹ-AGGTCTAGAGCGGTTCAGGAGCTTGATTCCTA-3ʹ; Rab6 Q72L-Rv: 5ʹ-TGAACCGCTCTAGACCTGCTGTGTCCCATAATTGC-3ʹ; Rab6 T27N-Fw: 5ʹ-GCGTTGGAAAGAACTCTTTGATCACCAGATTCATGTATGA-3ʹ; Rab6 T27N-Rv: 5ʹ-AAGAGTTCTTTCCAACGCTTTGCTCCCCCAGGA-3ʹ.

### Mice

Female 6- to 8-week-old C57BL/6 J mice were purchased from the Experimental Animal Center of Zhengzhou University (Zhengzhou, China) and maintained in a specific pathogen-free animal facility according to the Guide for the Care and Use of Laboratory Animals and related ethical regulations established at Henan Agricultural University.

### Cell viability analysis

Cell viability was assessed using a Cell Counting Kit-8 (CCK-8, GK3607, DingGuo). Cells were seeded in 96-well plates at a density of 1 × 10^4^ per well for 24 h. The next day, CCK-8 solution (10 μL) was added to each well, followed by incubation at 37 ℃ for 3 h. The absorbance was then determined at 450 nm with a microplate reader (Varioskan Flash, Thermo Fisher Scientific).

### Immunoblotting analysis

The cells were lysed in RIPA buffer (P0013B, Beyotime Biotechnology) supplemented with a protease and phosphatase inhibitor cocktail (HY-K0010 and HY-K0022, MedChemExpress). The protein concentration was determined using a BCA Protein Assay Kit (BCA01, DingGuo). The protein samples were separated by SDS‒PAGE and transferred to a membrane. The membrane was incubated with 5% skim milk (A600669, Sangon) for 1 h at room temperature. The membrane was then incubated with the primary antibody overnight at 4 ℃, followed by incubation with the appropriate horseradish peroxidase-conjugated secondary antibodies at room temperature for 1 h. Luminata Crescendo Western HRP substrate (WBLUR0500, Millipore) was used to visualize the immunoblotting results via a GE AI600 imaging system.

### qRT‒PCR analysis

Total RNA was extracted using TRIzol Reagent (TaKaRa) and reverse-transcribed with a PrimeScript RT reagent kit (RR047A, TaKaRa). qRT‒PCR was performed on a QuantStudio 6 Flex Real-Time PCR System using SYBR Premix ExTaq (TaKaRa). The data were normalized to the expression of the control gene *ACTB*. The transcripts were quantified using the 2^−ΔΔCt^ method. The following primers were used for qRT‒PCR analysis: porcine *ACTB*-Fw: 5ʹ-GCACAGAGCCTCGCCTT-3ʹ; porcine *ACTB*-Rv: 5ʹ-CCTTGCACATGCCGGAG-3ʹ; PRV *gH*-Fw: 5ʹ-CTCGCCATCGTCAGCAA-3ʹ; PRV *gH*-Rv: 5ʹ-GCTGCTCCTCCATGTCCTT-3ʹ; porcine *RAB6*-Fw: 5ʹ-GAGCGTACACCTAACCACCC-3ʹ; porcine *RAB6*-Rv: 5ʹ-GCGTCTTCCTGGTTTAGCCT-3ʹ; mouse *ACTB*-Fw: 5ʹ- CCCCATTGAACATGGCATTG-3ʹ; mouse *ACTB*-Rv: 5ʹ-ACG ACCAGAGGCATACAGG-3ʹ; mouse *Rab6*-Fw: 5ʹ-GACGTCCTTGATCACCCGAT-3ʹ; and mouse *Rab6*-Rw: 5ʹ-GCAGCAGAGTCACGGATGTA-3ʹ.

### Immunofluorescence staining analysis

Cells grown on climbing tablets were fixed with 4% paraformaldehyde in PBS at room temperature for 30 min. The cells were then permeabilized with 0.1% Triton X-100 for 10 min. Afterwards, they were incubated with PBS containing 10% FBS for 1 h at room temperature. The cells were washed with PBS and then incubated with the appropriate Alexa Fluor-conjugated secondary antibodies for 1 h at room temperature. Finally, the cells were mounted in ProLong Diamond with DAPI (P36971, Invitrogen). Images were captured using a Zeiss LSM 800 confocal microscope.

### RNA interference

Short hairpin RNAs (scrambled: 5ʹ-GCCACAACGTCTATATCATGG-3ʹ; shRab6-1: 5ʹ-GGAGCTTGATTCCTAGCTACA-3ʹ; shRab6-2: 5ʹ-GCTTGATTCCTAGCTACATTC-3ʹ; shRab6-3: 5ʹ-GCTACATTCGTGACTCCACTG-3ʹ) were synthesized as double-stranded oligonucleotides, cloned and inserted into the pLKO.1 vector and co-transfected with the packaging plasmids pMD2.G (12259, Addgene) and psPAX2 (12260, Addgene) into HE293T cells. Lentiviruses were harvested at 48 h post-transfection and used to infect cells, which were then subjected to with puromycin (4 μg/mL) selection for 7 days. The knockdown efficiency was determined by qRT‒PCR or immunoblotting analysis.

### Plaque assay

Vero cells grown in 12-well plates were infected with serially diluted PRV-QXX sample. After 3–5 days of incubation, when cytopathic effects were observed in approximately 80% of the cells, the cells were stained with 1% crystal violet. The dye solution was then rinsed with distilled water, and the number of plaques (empty spots) was calculated.

### The 50% tissue culture infective dose (TCID_50_) assay

On Day 0, Vero cells were seeded in a 96-well plate at 1 × 10^4^ cells per well. On Day 1, the cells were inoculated with serially diluted viruses (dilutions ranging from 10^−1^ to 10^−12^) for 1 h at 37 ℃. The excess viral inoculum was removed by washing with PBS. Then, 200 μL of maintenance medium (DMEM with 2% FBS) was added to each well, and the cells were cultured for 3–5 days. The cells were observed daily for the expected cytopathic effect, and the TCID_50_ value was calculated using the Reed–Muench method.

### Viral attachment assay

The cells were incubated with PRV-QXX (MOI = 10) at 4 ℃ for 2 h. After three extensive washes with ice-cold PBS, a viral attachment assay was performed via qRT‒PCR analysis of the number of PRV genome copies on the PM.

### Viral entry assay

The cells were incubated with PRV PRV-QXX (MOI = 10) at 4 ℃ for 2 h. Then, the cells were extensively washed with ice-cold PBS three times and incubated at 37 ℃ for 10 min to allow entry. After washing with trypsin (1 mg/mL) to remove the residual virions on the PM, viral entry was detected via qRT‒PCR analysis of viral genome copy numbers in the cells.

### Viral assembly assay

PK-15 cells were infected with PRV-QXX (MOI = 5). The assembly efficiency of the virus in the supernatant was determined by comparing the infection titre (TCID_50_/mL) with the total genomic equivalent of PRV (gE).

### Co-IP assay

Cells were harvested and lysed in 1 mL of RIPA buffer and clarified by centrifugation at 16 000 × *g* for 10 min at 4 ℃. Next, 900 μL aliquots were incubated with 40 μL of anti-FLAG M2 Affinity Gel (A2220, Sigma) for 4 h at 4 ℃. The beads were washed three times with lysis buffer and eluted with SDS sample buffer by boiling for 10 min before immunoblotting.

### Statistical analysis

The data are representative of at least three independent experiments for quantitative analysis and are expressed as the means ± standard errors of the means. All the data were analysed with Prism 7 software (GraphPad Software, Inc.) via two-tailed Student’s *t* tests, and *P* < 0.05 was considered to indicate statistical significance.

## Results

### PRV infection upregulates Rab6 expression

**Figure 1:**
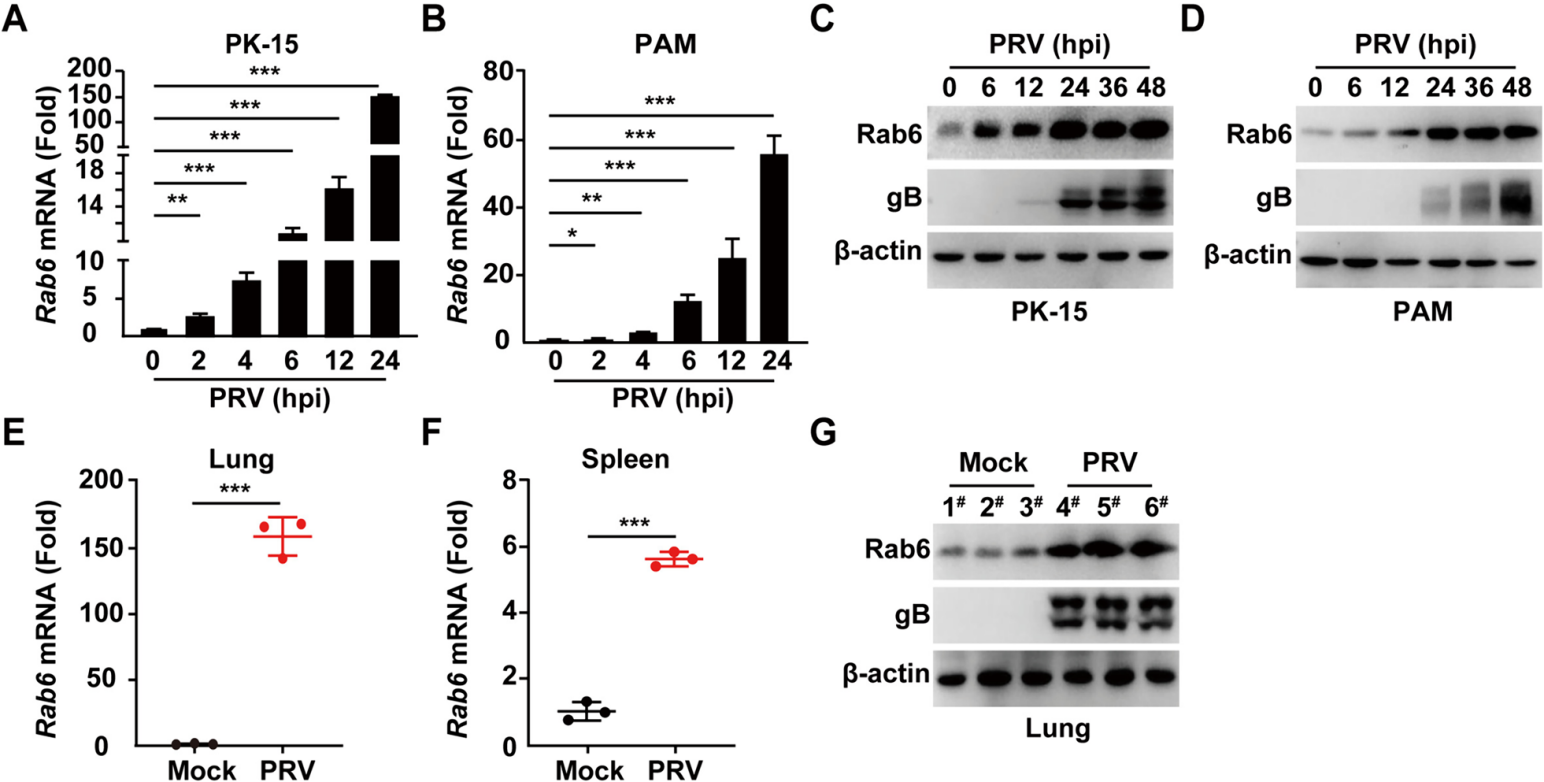
**PRV infection stimulates Rab6 expression. A** and **B** PK-15 cells and PAMs were infected with PRV-QXX (MOI = 1) for 24 h. Rab6 mRNA levels were evaluated via qRT‒PCR analysis. **P < 0.01, ***P < 0.001. **C** and **D** PK-15 cells and PAMs were infected with PRV-QXX (MOI = 1) for 24 h. The protein levels of Rab6 and PRV gB were evaluated by immunoblotting analysis. **E** and **F** Mice were either mock infected or intranasally infected with PRV-QXX (5 × 10^3^ TCID_50_/50 μL per mouse) for 3 days. The mRNA levels of Rab6 in the lungs and spleen were analysed by qRT‒PCR (*n* = 3 per group). ***P < 0.001. **G** Mice were treated as described in (**E**). The protein levels of Rab6 and PRV gB in the lungs were assessed by immunoblotting analysis (*n* = 3 per group).

To explore the role of Rab6 during PRV infection, we conducted a comprehensive analysis of Rab6 expression following PRV exposure in vitro. Cultured cells were infected with the PRV-QXX strain and incubated for various durations, ranging from 0 to 24 h; subsequently, we quantified the Rab6 mRNA and protein levels in these cells. Notably, PRV infection significantly increased Rab6 mRNA levels in both PK-15 (porcine kidney) and porcine alveolar macrophage (PAM) cells (Figures 1A and B). This increase in mRNA expression was paralleled by a corresponding increase in Rab6 protein levels in PK-15 and PAM cells post-infection, suggesting consistent upregulation of both Rab6 mRNA and protein expression (Figures 1C and D).

Extending our investigation to a physiological context, we sought to verify whether PRV infection similarly upregulates Rab6 expression in vivo. To this end, mice were either left as mock-infected controls or intranasally inoculated with the PRV-QXX strain, and their tissues were harvested after 3 days for analysis. Using quantitative real-time polymerase chain reaction (qRT‒PCR), we measured Rab6 mRNA levels in both the lungs and spleens, while Rab6 protein levels were exclusively assessed in lung tissues through immunoblotting analysis. Our findings revealed a significant increase in Rab6 expression in the tissues of PRV-infected mice compared to mock-infected controls (Figures 1E–G). Collectively, these observations strongly implied that PRV infection facilitates the upregulation of Rab6 both in vitro and in vivo.

### Rab6 overexpression promotes PRV infection

**Figure 2:**
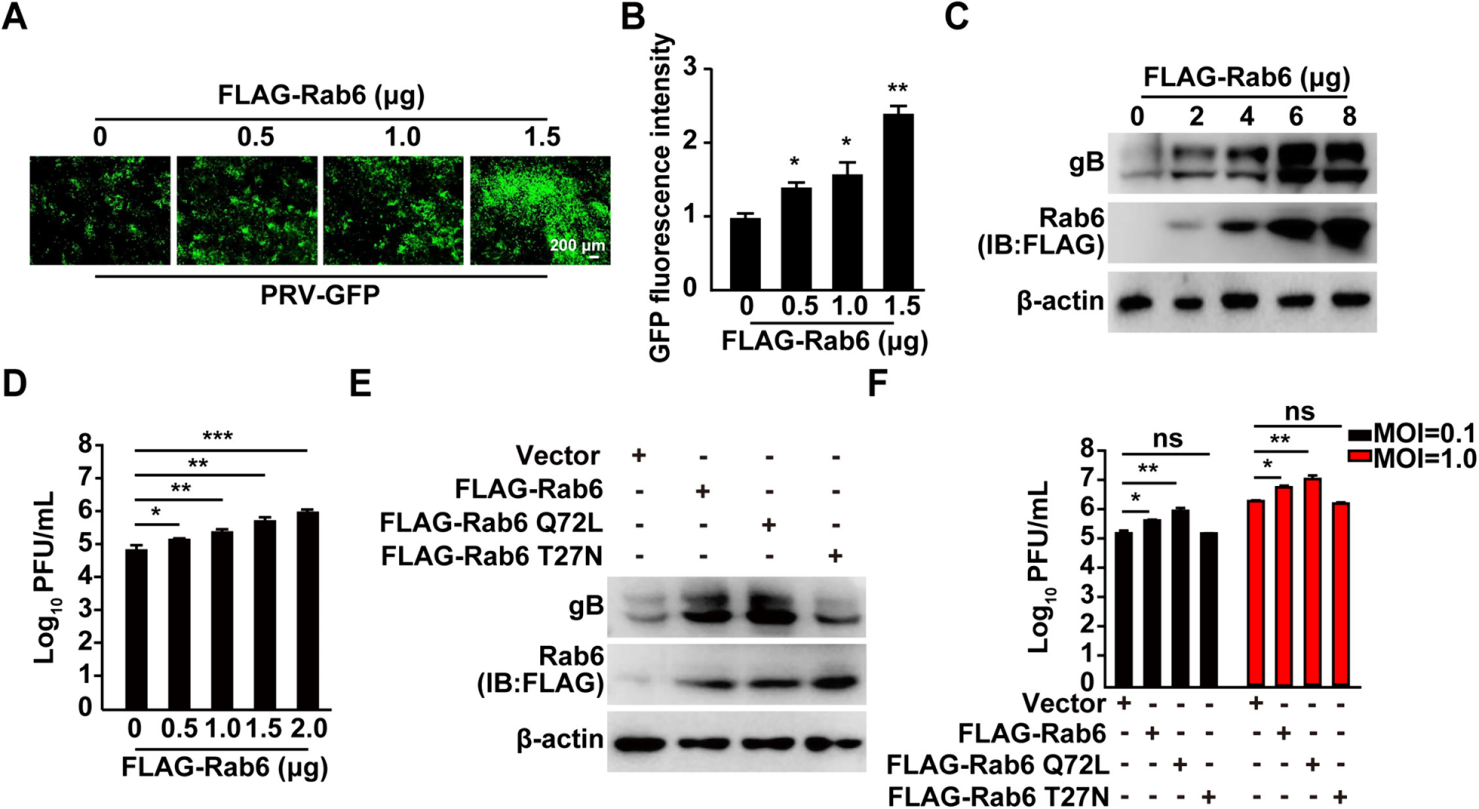
**Rab6 overexpression promotes PRV infection**. **A** PK-15 cells were transfected with the FLAG-Rab6 plasmid (0–1.5 μg) supplemented with 1.5 μg of p3 × FLAG-CMV-10 for 12 h and then infected with PRV-GFP (MOI = 0.001) for 24 h. Viral replication was assessed using fluorescence microscopy. Scale bar: 200 μm. **B** The relative fluorescence intensity in (**A**) was analysed using ImageJ. *P < 0.05, **P < 0.01. **C** PK-15 cells were transfected with the FLAG-Rab6 plasmid (0–8 μg) supplemented with p3 × FLAG-CMV-10 to a total of 8 μg for 12 h and then infected with PRV-QXX (MOI = 1) for 24 h. The protein levels of PRV gB and FLAG-Rab6 were assessed by immunoblotting analysis. **D** PK-15 cells were transfected with the FLAG-Rab6 plasmid (0–2 μg) supplemented with p3 × FLAG-CMV-10 to a total of 2 μg for 12 h and then infected with PRV-QXX (MOI = 1) for 24 h. Viral titres were determined via plaque assays. *P < 0.05, **P < 0.01, ***P < 0.001. **E** PK-15 cells were transfected with the FLAG-CMV-10 (vector) or the FLAG-Rab6, FLAG-Rab6 Q72L, or FLAG-Rab6 T27N plasmids for 12 h and then infected with PRV-QXX (MOI = 1) for 24 h. The protein levels of PRV gB and FLAG-Rab6 were assessed by immunoblotting analysis. **F** PK-15 cells were transfected with p3 × FLAG-CMV-10 (vector) or the FLAG-Rab6, FLAG-Rab6 Q72L, or FLAG-Rab6 T27N plasmids for 12 h and then infected with PRV-QXX (MOI = 0.1 and 1) for 24 h. Viral titres were determined by plaque assays. *P < 0.05, **P < 0.01. ns: not significant.

To investigate the role of Rab6 in PRV proliferation, we engineered a plasmid that encodes FLAG-tagged Rab6. PK-15 cells were transfected with various concentrations of this FLAG-Rab6 construct or a control vector for 24 h before being infected with PRV-GFP. Using fluorescence microscopy and subsequent analysis of fluorescence intensity, we observed a notable increase in the GFP signal in cells overexpressing FLAG-Rab6, indicating that Rab6 overexpression facilitated PRV-GFP infection (Figures 2A and B). Furthermore, to understand the impact of Rab6 overexpression on the expression of PRV glycoprotein B (gB), we conducted immunoblotting analyses. The results showed a marked upregulation of PRV gB expression in cells overexpressing FLAG-Rab6 (Figure 2C). Additionally, plaque assays demonstrated that Rab6 overexpression significantly increased the generation of viable viral progeny (Figure 2D).

To further elucidate the mechanistic role of Rab6, we generated mutants that mimic its constitutively active (GTP-bound) state, Rab6 Q72L, and its dominant-negative (GDP-bound) state, Rab6 T27N. PK-15 cells were transfected with the control vector, FLAG-Rab6 Q72L, or FLAG-Rab6 T27N. Notably, the overexpression of Rab6 Q72L led to a substantial increase in PRV gB expression, highlighting its ability to promote PRV infection (Figure 2E). In contrast, overexpression of Rab6 T27N did not significantly alter PRV gB expression levels, as evidenced by immunoblotting analyses (Figure 2E). Correspondingly, plaque assays revealed that Rab6 Q72L overexpression significantly enhanced virus proliferation, whereas Rab6 T27N overexpression had no appreciable impact on viral growth (Figure 2F). These findings indicated that the overexpression of Rab6, particularly in its active form, accelerates PRV proliferation.

### Rab6 knockdown inhibits PRV infection

**Figure 3:**
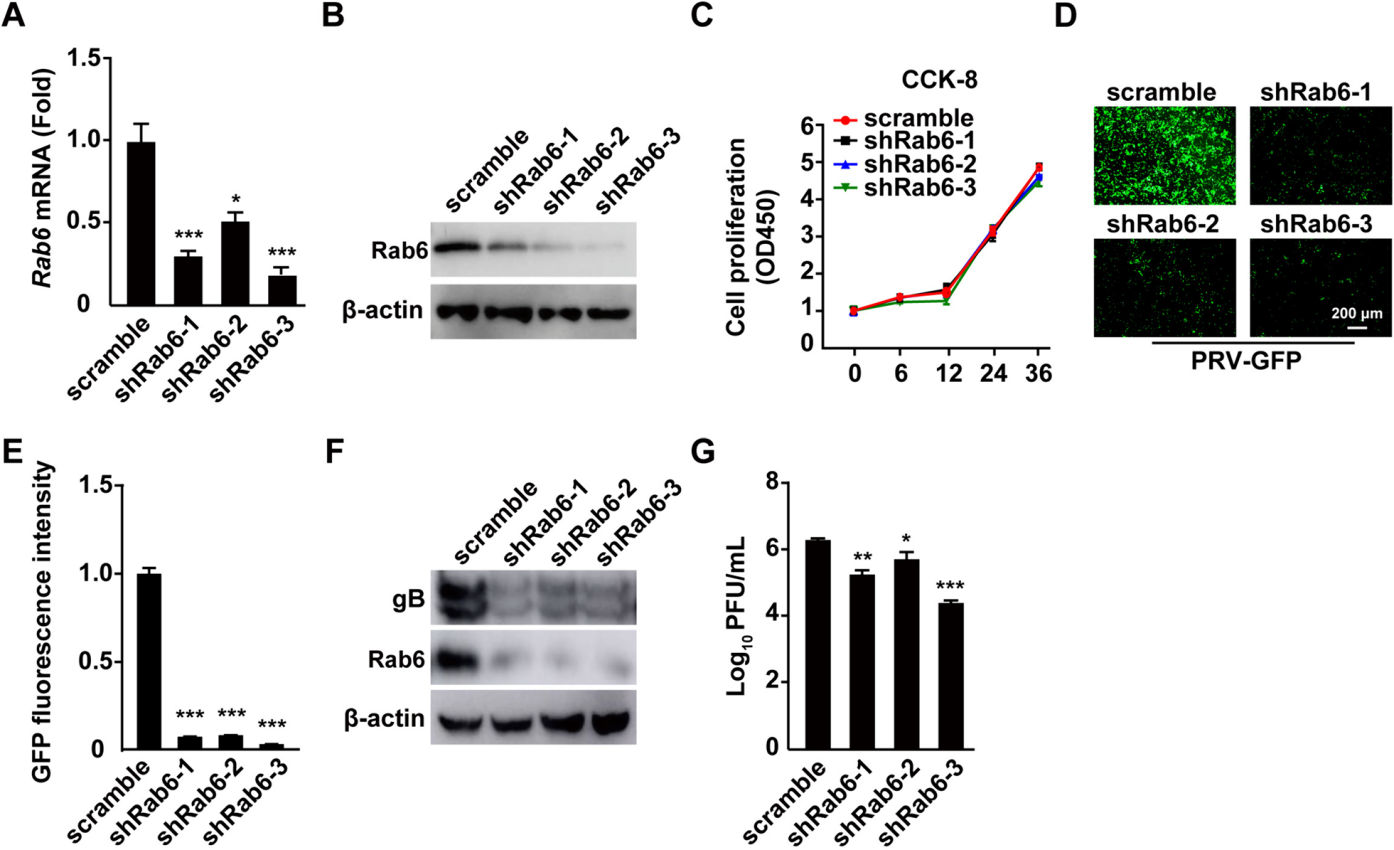
**Rab6 knockdown inhibits PRV infection**.**A** and **B** The levels of Rab6 mRNA and protein in PK-15 cells transfected with scrambled, shRab6-1, shRab6-2, or shRab6-3 were assessed by qRT‒PCR and immunoblotting analysis, respectively. *P < 0.05, ***P < 0.001. **C** The viability of PK-15 cells transfected with scrambled, shRab6-1, shRab6-2, or shRab6-3 was assessed by a CCK-8 assay. **D** PK-15 cells transfected with scrambled, shRab6-1, shRab6-2, or shRab6-3 and infected with PRV-GFP (MOI = 0.001) for 24 h were observed under a fluorescence microscope to analyse viral proliferation via GFP expression. Scale bar: 200 μm. **E** The relative fluorescence intensity in (**D**) was analysed using ImageJ. **P < 0.01, ***P < 0.001. **F** PK-15 cells transfected with scrambled, shRab6-1, shRab6-2, or shRab6-3 and infected with PRV-QXX (MOI = 1) for 24 h. The protein levels of PRV gB and Rab6 were assessed by immunoblotting analysis. **G** PK-15 cells were transfected with scrambled, shRab6-1, shRab6-2, or shRab6-3 and infected with PRV-QXX (MOI = 1) for 24 h. Viral titres were determined by plaque assays. *P < 0.05, **P < 0.01, ***P < 0.001.

To further substantiate the involvement of Rab6 in PRV infection, we reduced Rab6 expression via short hairpin RNA (shRNA)-mediated RNA interference. Both qRT‒PCR and immunoblotting confirmed the effective knockdown of Rab6 expression by three specific Rab6-targeting shRNAs in PK-15 cells (Figures 3A and B). Importantly, this reduction in Rab6 did not adversely affect the viability of the cells (Figure 3C), ensuring that the observed effects on viral infection were not secondary to cellular health.

Subsequently, we explored the impact of Rab6 depletion on PRV propagation. PK-15 cells transfected with either scrambled shRNA or one of the Rab6-specific shRNAs (shRab6-1, shRab6-2, or shRab6-3) were infected with PRV-GFP. Assessment of viral proliferation via fluorescence microscopy revealed a marked decrease in fluorescence intensity in Rab6-depleted cells compared to control cells, indicating that Rab6 knockdown hampers PRV-GFP replication (Figures 3D and E). To provide further insights into viral replication post-Rab6 knockdown, we measured the level of the PRV gB protein by immunoblotting analysis. The results demonstrated significantly lower gB protein levels in Rab6-knockdown cells than in control cells (Figure 3F).

Moreover, the viral titre, which was assessed after infection of cells with PRV-QXX, was substantially lower in cells in which Rab6 was depleted than in cells transfected with the scrambled shRNA (Figure 3G). These results indicated that Rab6 plays a pivotal role in facilitating PRV proliferation.

### Rab6 is involved in PRV assembly and egress

**Figure 4:**
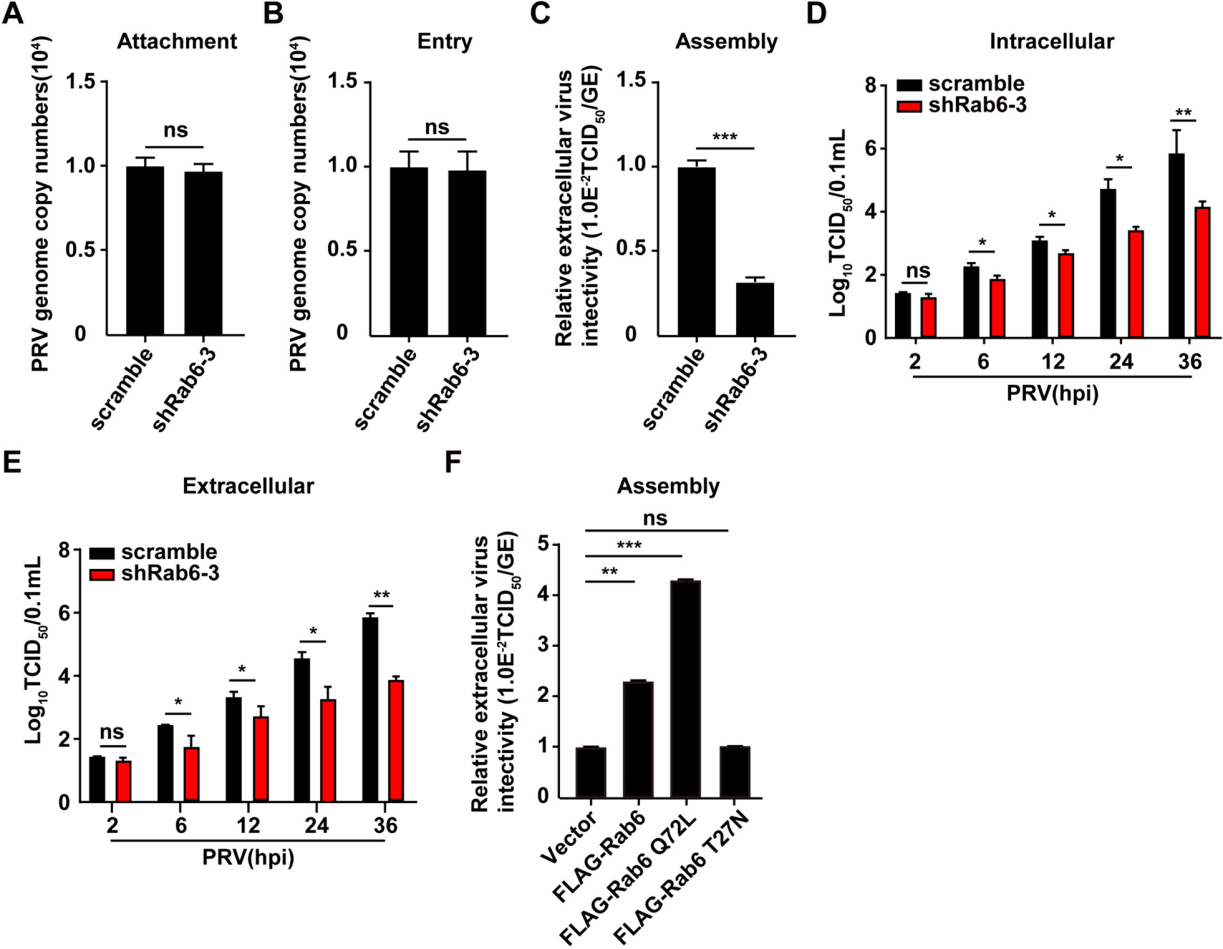
**Rab6 is involved in PRV assembly**. **A** PK-15 cells transfected with scrambled or shRab6-3 were incubated with PRV (MOI = 10) at 4 ℃ for 2 h. After the cells were washed three times with ice-cold PBS, viral attachment was assessed by qRT‒PCR analysis of genome copy numbers at the PM. *ns* not significant. (**B**) PK-15 cells transfected with scrambled or shRab6-3 were incubated with PRV (MOI = 10) at 4 ℃ for 2 h, extensively washed with ice-cold PBS three times, and then incubated at 37 ℃ for 10 min to allow entry. After washing with trypsin (1 mg/mL) to remove residual virions on the PM, viral entry was detected via qRT‒PCR analysis of viral genome copy numbers in the cells. *ns* not significant. (**C**) PK-15 cells transfected with scrambled or shRab6-3 were infected with PRV (MOI = 5) for 24 h. The efficiency of viral assembly in the supernatants was determined by comparing the infectious titres (TCID_50_ per millilitre) with the total number of PRV genome equivalents (GE). **P < 0.01. (**D** and **E**) PK-15 cells transfected with scrambled or shRab6-3 were infected with PRV-QXX (MOI = 5) had their extracellular (**D**) and intracellular (**E**) viruses harvested and subjected to a TCID_50_ assay to determine viral titres at 2–36 h post-infection (hpi). *P < 0.05. (**F**) PK-15 cells were transfected with p3 × FLAG-CMV-10 (vector), FLAG-Rab6, FLAG-Rab6 Q72L, or FLAG-Rab6 T27N for 12 h and then infected with PRV-QXX (MOI = 5) for 24 h. The efficiency of viral assembly in the supernatants was determined by comparing the infectious titres (TCID_50_ per millilitre) with the total number of PRV genome equivalents (GE). ***P < 0.001. ns: not significant.

To deepen our understanding of the impact of Rab6 on the PRV life cycle, we meticulously dissected the stages in which Rab6 plays a pivotal role. The initial phase of viral infection involves the attachment of the virus to host cells. To investigate this possibility, we exposed both scrambled-transfected and shRab6-1-transfected PK-15 cells to PRV-QXX at 4 ℃ for 2 h, which facilitated viral attachment without entry. After thorough washing with ice-cold phosphate-buffered saline (PBS) to remove unbound viruses, the extent of viral attachment on the cell surface was quantified by qRT‒PCR analysis, which measured viral genome copy numbers on the cell surface. Next, the cells were incubated at 37 ℃ for 10 min to permit viral entry. We then used a 1 mg/mL trypsin wash to eliminate any remaining surface-bound virions. Again, qRT‒PCR analysis of viral genome copy numbers within the cells was used to assess the efficiency of viral entry. These results indicated that Rab6 depletion does not significantly influence PRV attachment or entry into host cells (Figures 4A and B).

Proceeding to the viral assembly phase, we observed a noticeable decrease in virus assembly efficiency following Rab6 knockdown, which emphasized the essential role of Rab6 in this process (Figure 4C). Additionally, to assess the infectivity of the assembled viral progeny, we performed a TCID_50_ assay on both intracellular and extracellular PRV. Notably, both the intracellular and extracellular PRV titres were reduced 2 h post-infection in Rab6-depleted cells (Figures 4D and E).

To further elucidate the influence of Rab6 on PRV assembly and egress, we transfected PK-15 cells with vectors encoding FLAG-Rab6, the constitutively active mutant FLAG-Rab6 Q72L, or the dominant-negative mutant FLAG-Rab6 T27N or an empty vector. Subsequent viral infection and analysis revealed that the overexpression of FLAG-Rab6 significantly enhanced PRV assembly and egress compared to the empty vector control (Figure 4F). Intriguingly, while FLAG-Rab6 alone enhanced PRV assembly and egress efficiency, the overexpression of FLAG-Rab6 Q72L showed an even greater enhancement of this effect. In contrast, the overexpression of FLAG-Rab6 T27N did not have any impact on PRV assembly or egress compared to the control group (Figure 4F). Collectively, these findings demonstrated that Rab6 is critical for PRV assembly and egress.

### Rab6 interacts with PRV gB and gE

Current evidence supports the role of Rab6 in influencing the assembly stages of viruses. It is well established that the glycoproteins of herpes viruses play a pivotal role in the assembly and subsequent release of progeny viruses [20]. Specifically, the viral glycoproteins are packaged within the secondary envelope of vesicles emanating from the trans-Golgi network during the virus assembly process [21].

**Figure 5:**
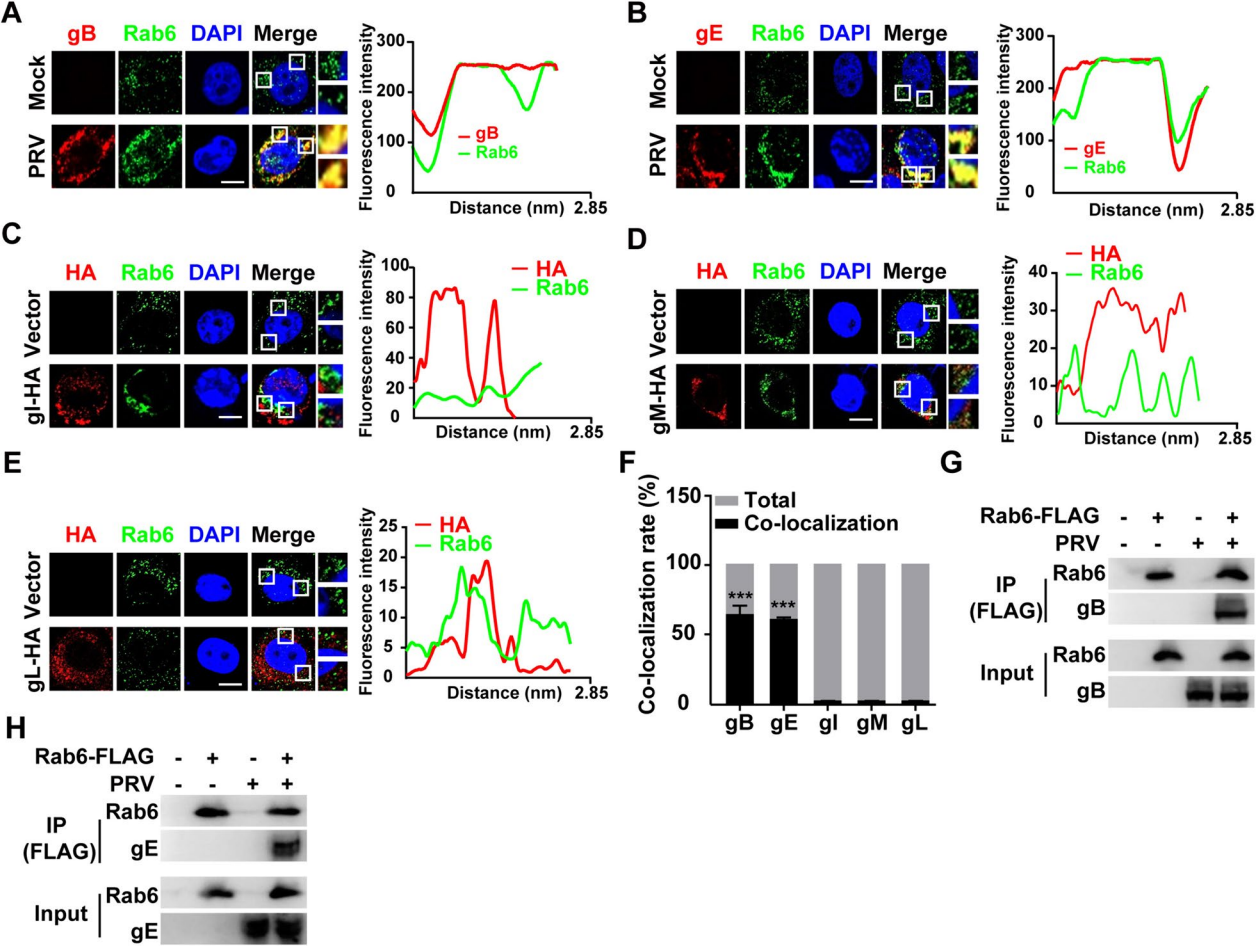
**Rab6 interacts with PRV gB and gE**. **A** PK-15 cells were mock-infected or infected with PRV-QXX (MOI = 1) for 24 h. Rab6 (green) and gB (red) were detected by immunofluorescence analysis. The graphs depict the application of ImageJ software to conduct line scan analysis on the image and to analyse the relative position of the two markers. Scale bar: 10 μm. **B** PK-15 cells were mock-infected or infected with PRV-QXX (MOI = 1) for 24 h. Rab6 (green) and gE (red) were detected by immunofluorescence analysis. The graphs depict the application of ImageJ software to conduct line scan analysis on the image and to analyse the relative position of the two markers. Scale bar: 10 μm. (**C**–**E**) PK-15 cells were transfected with HA-tagged gI (**C**), gM (**D**), or gL (**E**) plasmids for 24 h. The colocalization of Rab6 with gI, gM, and gL was determined by immunofluorescence analysis. Scale bar: 10 μm. The graphs depict the application of ImageJ software to conduct line scan analysis on the image and to analyse the relative position of the two markers. Scale bar: 10 μm. **F** Rab6 colocalization was measured in cells infected with PRV, uninfected cells, and cells transfected with either empty or virus-associated proteins (*n* = 30). (G and H) PK-15 cells were transfected with p3 × FLAG-CMV-10 (vector) or FLAG-Rab6 for 12 h and then infected with PRV-QXX (MOI = 1) for 24 h. The interaction of Rab6 with gB (**F**) and gE (**G**) was analysed by co-IP analysis.

To investigate the correlation between Rab6 and the PRV glycoproteins gB and gE, we investigated their colocalization. This study focused on analysing the colocalization of gB and gE monoclonal antibodies with Rab6 after PK-15 cells were infected with PRV. The findings demonstrated significant colocalization of these two glycoproteins with Rab6 (Figures 5A and B), suggesting a direct interaction during the assembly and egress process. Additionally, we introduced plasmids carrying HA-tagged gI, gM, and gL into the cells and utilized immunofluorescence to monitor their localization. In contrast to gB and gE, neither gI nor gL colocalized with Rab6 (Figures 5C–E). However, it is important to note that gI and gL function in heterodimeric complexes with gE and gH, respectively. In the absence of gE, gI mislocalizes, while gL is not membrane associated and is secreted when gH is not coexpressed. Although there was some colocalization between gM and Rab6, this colocalization was not as noticeable as that observed for gB or gE. Additionally, a more in-depth statistical analysis of colocalization revealed that Rab6 colocalized with PRV gB and gE (Figure 5F). This indicated the selective interaction of Rab6 with specific glycoproteins.

For a more conclusive demonstration of the interaction between Rab6 and the glycoproteins gB and gE of PRV, we employed a coimmunoprecipitation (Co-IP) assay. We performed this assay on PK-15 cells that had been either mock-infected or infected with PRV. The results confirmed that PRV gB and PRV gE coimmunoprecipitated with Rab6 (Figures 5G and H). In conclusion, our investigations revealed the involvement of Rab6 in PRV assembly and egress, primarily through its association with the glycoproteins gB and gE.

## Discussion

Host cells are essential for the completion of the life cycle of viruses [22]. Viruses are composed of either an RNA or DNA genome encased in a protein capsid [23]. Certain viruses also possess a lipid bilayer derived from the cellular membranes of their host. To replicate, viruses must exploit host cell systems to produce new RNA, DNA, proteins, and lipid envelopes [1, 24]. Although virus life cycles may vary, they all share five steps: entry into the cell, translation of viral proteins, replication of the genome, assembly of the viral particle, and exit from the cell [25]. Enveloped viruses typically rely on the host's vesicular trafficking pathways to transport their structural proteins and genetic material to sites of replication, assembly, and budding [11]. Rab GTPases, the largest family of small GTPases, include nearly 70 members involved in vesicular transport [26]. The Golgi Rab group predominantly consists of the Rab6 protein, which belongs to the Rab6 subfamily [27, 28]. The Rab family clearly plays a pivotal role in viral infection processes. The Golgi apparatus, positioned at the core of the internal membrane system of animal cells, is crucial for the post-translational modification of proteins and the organization of secretory pathway transport. Rab6, a crucial protein in the Rab family, plays a role in retrograde transport within the Golgi apparatus. Additionally, Rab6 can be found on secretory vesicles that travel from the Golgi to the plasma membrane, a process that is particularly important for viral egress [29–32]. This study highlights the significance of Rab6 in PRV infection, as it acts as a cofactor to facilitate virus assembly and egress, thereby aiding in PRV replication.

Investigations have confirmed that Rab proteins and their effector components are essential for virus proliferation. For example, Rab2 has been shown to promote the proliferation of classical swine fever virus [33, 34], and Rab11a has been shown to enhance the replication of PRRSV in the host organism by regulating autophagy [35]. Our results demonstrated that an increase in Rab6 expression was linked to PRV infection and that overexpression of Rab6 enhanced PRV proliferation. Conversely, Rab6 knockdown inhibited PRV proliferation. To investigate the effect of Rab6 on PRV proliferation, we examined the life cycle of the virus, since the exact stage at which Rab6 influences the cell cycle remains unknown. Researchers have studied Rab proteins and demonstrated that viral glycoproteins first travel to the plasma membrane and then are endocytosed with the help of Rab5 and Rab11. It is within these endocytic membranes where secondary envelopment takes place [36, 37]. Research has shown that Rab6 plays a role in both the formation and release of HSV-1 and is also associated with VZV particles [38, 39]. Our research revealed that knockdown of Rab6 had no effect on the adsorption or entry of PRV. However, experiments on virus assembly and egress have shown that inhibiting Rab6 hinders this process. Additionally, overexpression of Rab6 in PK-15 cells facilitated PRV assembly and egress. These findings indicate the significant role of Rab6 in the assembly and egress of PRV.

The specific molecular processes that PRV employs to exploit the cell's transportation system during morphogenesis remain unclear. Further exploration is required to understand the details regarding the secondary envelopment and exit of the virus. The final stages of viral assembly involve the budding of trans-Golgi network-derived vesicles coated with viral glycoproteins and tegument proteins, an event known as secondary envelopment [40, 41]. Recent investigations have provided evidence that enveloped viruses utilize Rab proteins for morphogenesis and egress pathways. For instance, research has shown that both influenza A and respiratory syncytial virus employ the Rab11a pathway for enveloping and budding [42], while Rab27a influences HSV-1 viral assembly by interacting with the glycoprotein gB [14]. Rab6 is known to be involved in the transport and release of PRV or alpha herpesvirus particles from Rab6 vesicles, as indicated by previous research [43–45]. Therefore, we investigated how viral glycoproteins interact with Rab6. Our findings indicated that Rab6 was linked to PRV gB and gE; however, Rab6 did not colocalize with gI or gL. There was some degree of colocalization between gM and Rab6. Co-IP confirmed the interaction of Rab6 with both gB and gE. In summary, our research suggested that Rab6 could be a beneficial host factor in PRV replication and thus a valuable target for developing drugs and vaccines to inhibit PRV replication.

## Data Availability

All the data and materials generated for this study are included in the article.
